# Dominant Effect of Ecological Restoration on Microbial Carbon Cycle in Plant Rhizosphere of Mining Areas

**DOI:** 10.3390/microorganisms14081713

**Published:** 2026-08-04

**Authors:** Yabo Pan, Hengfang Wang, Li Sun, Haishan Huang, Wenyan Liang, Honglin Liu

**Affiliations:** 1 College of Ecology and Environment, Xinjiang University, Urumqi 830046, China; 107552405037@stu.xju.edu.cn (Y.P.); sunli12201231@163.com (L.S.); 2 Key Laboratory of Oasis Ecology of Ministry of Education, Xinjiang University, Urumqi 830046, China; 3 Xinjiang Sayr Energy Co., Ltd., Hoboksar Mongol Autonomous County 834400, China; 18124313970@163.com (H.H.); xksenyghhbb@126.com (W.L.); 4 School of Geology and Mining Engineering, Xinjiang University, Urumqi 830047, China; liuhonglin@xju.edu.cn

**Keywords:** mining area restoration, metagenomics, carbon cycling, carbon fixation genes, microorganisms

## Abstract

Rhizosphere microorganisms play critical roles in biogeochemical processes including carbon cycling; however, their linkages to carbon-cycling functions under distinct mine restoration approaches remain unclear. In this study, we compared natural and artificial vegetation restoration via metagenomic sequencing to characterize rhizosphere microbial communities and carbon-cycling functional genes. Artificial restoration (AR) decreased soil electrical conductivity (EC) and salt content (SC) while increasing total phosphorus and available phosphorus by 12.33% and 16.44%, respectively. AR also elevated the relative abundances of genes responsible for degrading aromatic compounds, lignin and starch, along with genes participating in carbon-fixation pathways. Taxa of the Actinomycetia, Chloroflexi, and Solirubrobacterales served as the primary contributors to genes encoding 2-isopropylmalate synthase, α-glucosidase, malate synthase, and α-mannosidase, as well as carbon-fixation-related genes, including *aconitate hydratase (ACO)*, *methylmalonyl-CoA mutase subunit (E5.4.99.2A)*, *pyruvate orthophosphate dikinase (ppdK)*, and *phosphoenolpyruvate carboxylase (ppc)*. Moreover, the relative abundances of carbon-fixation genes exhibited significant positive correlations with EC, SC, nitrate nitrogen (NO_3_^−^-N) and ammonium nitrogen (NH_4_^+^-N). Overall, microorganisms in AR soils hold relatively high genetic potential for carbon sequestration and decomposition. Such rhizosphere carbon-cycling functions are jointly shaped by restoration strategies and vegetation community composition, and our findings offer key theoretical support for mine ecological restoration.

## 1. Introduction

The extraction of resources in mining areas provides essential support for national energy security and economic development. However, mining activities often cause substantial ecological disturbances, including soil degradation and vegetation loss [1]. Vegetation destruction can alter microbial community diversity and reduce ecosystem carbon stocks, thereby exacerbating global climate change [2]. Therefore, effective post-mining ecological restoration strategies are critical for accelerating ecosystem recovery in mining-affected areas.

Within the broader framework of ecological restoration, vegetation restoration in mining areas is commonly achieved through two main pathways: natural recovery and artificial restoration [3]. The different restoration strategies exhibit unique nutrient cycling patterns and impacts on soil functions, leading to the formation of distinct soil microbial communities. Microorganisms, as the core mediators of the “soil–plant” interaction network [4,5], dominate the material cycling and energy flow in mining areas. They participate in carbon transformation and storage through organic matter decomposition and carbon fixation, influencing the soil carbon cycling process [6,7,8]. Previous studies have shown that natural recovery can improve soil physicochemical properties and enzyme activities in mining areas and may enhance microbial carbon fixation potential by modifying environmental conditions [9]. In the context of vegetation restoration in mining areas, natural recovery utilizes the self-healing capacity of ecosystems to restore the ecological functions of degraded lands and may contribute to maintaining ecological stability and local biodiversity. However, this is usually a long-term process. In contrast, artificial restoration can accelerate the ecological sequence, shorten the recovery cycle, and promote faster vegetation restoration [10]. Accordingly, the role of artificial restoration in enhancing carbon cycling in mining areas has received increasing attention [11]. Related studies have shown that after vegetation restoration in mining areas, the recovery of ecosystem functions and carbon sequestration capacity is greatly enhanced [12]. Other work has further demonstrated that different vegetation types can have contrasting effects on soil chemical properties and the abundance of functional genes associated with carbon cycling in mining soils [13]. Additionally, artificial restoration can markedly increase microbial abundance and diversity, enhancing gene richness, and contribute to microbial multifunctionality in degraded mining ecosystems [14]. Restoration can also increase soil nutrient availability; for example, the mass fractions of total phosphorus (TP) and total nitrogen (TN) have been reported to increase by 1.44–1.96 times and 1.56–2.11 times, respectively, after restoration [15]. These findings indicate that artificial restoration can rapidly improve soil physicochemical conditions, thereby benefiting the soil environment and facilitating ecosystem recovery in mining areas [16].

Vegetation restoration in mining areas can improve soil environmental quality and enhance ecosystem carbon-sink functions [17]. However, although previous studies have documented changes in soil physicochemical properties, microbial diversity, and carbon fixation following vegetation restoration, the microbial mechanisms governing carbon cycling under different restoration strategies remain insufficiently resolved [18,19]. In particular, most studies have focused on non-rhizosphere soil properties, vegetation development, or overall microbial community composition [20]. Comparatively few have directly compared natural recovery and artificial restoration within the same mining area from the perspective of rhizosphere microbial functional genes involved in carbon cycling. The rhizosphere is a critical interface for soil–plant–microbe interactions and plays an important role in regulating organic carbon decomposition and microbial carbon fixation [21]. Nevertheless, it remains unclear whether artificial restoration enhances the metagenomic potential for both carbon decomposition and carbon fixation compared with natural recovery, which microbial taxa primarily drive these functional differences, and which soil or plant factors are associated with the changes in carbon-cycling genes. This knowledge gap limits mechanistic understanding of how different restoration strategies regulate microbial-mediated carbon cycling and carbon fixation potential in mining ecosystems.

To address these gaps, this study compared natural recovery (NR) and artificial restoration (AR) in a coal mining area and investigated soil properties, rhizosphere microbial community structure, and microbial carbon-cycling functional genes using metagenomic sequencing. The specific objectives were to (1) evaluate differences in rhizosphere soil physicochemical properties and microbial community diversity between NR and AR; (2) compare the metagenomic potential for microbial carbon decomposition and carbon fixation under the two restoration strategies, focusing on genes associated with the degradation of starch, cellulose, hemicellulose, lignin, pectin, chitin, and aromatic compounds, as well as genes involved in major microbial carbon fixation pathways, including the Calvin cycle; and (3) identify the key microbial taxa and environmental factors associated with carbon-cycling functional genes. We hypothesized that, compared with NR, AR would improve rhizosphere soil conditions, increase microbial diversity, and enrich microbial taxa involved in carbon decomposition and carbon fixation. This study employs metagenomics to compare rhizosphere microbial carbon-cycling functional potential in mining soils under different vegetation restoration strategies, and provides theoretical support and references for optimizing restoration approaches to improve soil quality and carbon cycling functions in degraded mining ecosystems.

## 2. Materials and Methods

### 2.1. Study Site

The northern Yining mining district is located in the northern Ili Basin, Ili Kazakh Autonomous Prefecture, Xinjiang, China. The district covers approximately 555 km^2^ and spans 43°48′ N–44°14′ N, 80°41′ E–81°50′ E [22]. The region has a mean annual temperature of 2.9–9.4 °C, mean annual precipitation of 321.7 mm, and mean annual evaporation of 1495.84 mm. Evaporation far exceeds precipitation, rendering this study area a typical arid and semi-arid mining zone. Characterized by prolonged winters and summers, brief springs and autumns, and abundant sunshine, the area boasts ample light and thermal resources with a temperate continental climate [23]. Calcic gray soil dominates the study area, with moderate water erosion as the primary erosion pattern [24].

### 2.2. Experiment Design

In April 2020, experimental plots were deployed within a coal mining subsidence zone with homogeneous environmental backgrounds, including consistent soil type, terrain, mining disturbance legacy, and vegetation degradation severity. Two restoration strategies were compared: natural recovery (NR) and artificial restoration (AR). Three independent 5 m × 5 m replicate plots were established for each restoration regime, generating six experimental plots in total. Each plot was demarcated with perimeter markers to define independent experimental units. A 2 m wide buffer zone was retained between neighboring plots to mitigate cross-plot interference and extrinsic disturbances.

The NR plots were allowed to recover through passive natural succession, with *Bassia scoparia* and *Leucophylum frutescens* as the dominant plant species. In the AR plots, seeds of three native plant species—*Artemisia scoparia*, *Ceratocarpus arenariu*, and *Seriphidium kaschgaricum*—were sown in mid-April 2020. The seeds were purchased from Lvjing Ecology Co., Ltd. (Yili, Xinjiang, China), and all met the national Grade I seed-quality standards, with purity ≥ 98% and germination rate ≥ 90%. During the initial establishment phase, supplemental irrigation was applied for one month to enhance seed germination and seedling survival. Thereafter, no fertilization, soil amendment, or long-term irrigation was applied during the restoration period from 2020 to 2023.

### 2.3. Plant and Soil Samples Collection

Plant and soil samples were collected on 30 July 2023. To ensure spatial representativeness within each plot and minimize edge effects, a three-point diagonal random sampling design was used. In each 5 m × 5 m plot, a diagonal transect was established, and three sampling areas were positioned at the two ends of the diagonal and at the plot centre. All sampling points were located within the plot and at least 0.5 m from the plot boundary. In each sampling area, one healthy individual of each dominant plant species was randomly selected. Thus, three healthy individuals of each dominant species were sampled from each plot. Plant tissue samples were collected from the same individuals used for rhizosphere soil sampling. After collection, plant samples were oven-dried and ground to determine plant organic carbon, total nitrogen, total phosphorus, soluble sugar, and starch contents. These indicators were used for subsequent correlation analysis with carbon cycle genes. Rhizosphere soil was collected simultaneously with plant sampling. Loosely attached soil was gently removed from the root surface by shaking, and soil tightly adhering to the roots was collected using a sterile brush. The remaining thin layer of soil adhering to the root surface was then rinsed with sterile phosphate-buffered saline (PBS), and the resulting PBS wash suspension was collected in sterile 10 mL centrifuge tubes (Wuhan Servicebio Technology Co., Ltd., Wuhan, China). Samples were immediately frozen in liquid nitrogen and stored for subsequent DNA extraction and metagenomic sequencing. Rhizosphere soil collected from three individuals of the same plant species within the same plot was thoroughly homogenized to generate one composite rhizosphere soil sample. In total, 15 composite rhizosphere soil samples were obtained for metagenomic sequencing, including six samples from the natural recovery (NR) plots and nine samples from the artificial restoration (AR) plots. Non-rhizosphere soil was collected from the 0–20 cm soil layer using the same three-point sampling design. The collected non-rhizosphere soil was divided into two subsamples: one was stored at 4 °C for the determination of soil enzyme activities, whereas the other was air-dried, and passed through a 100-mesh sieve (Shaoxing Shangyu Hujiang Instrument Factory, Shaoxing, China) for the analysis of soil physicochemical properties.

### 2.4. Sample Analysis

This study assessed a suite of soil physicochemical and plant-tissue indicators. Soil variables included potential of hydrogen (pH), electrical conductivity (EC), salt content (SC), soil organic carbon (SOC), ammonium nitrogen (NH_4_^+^-N), nitrate nitrogen (NO_3_^−^-N), total phosphorus (TP), available phosphorus (AP), urease activity, and phosphatase activity. Plant-tissue variables included plant tissue organic carbon (POC), plant tissue total nitrogen (PTN), plant tissue total phosphorus (PTP), plant soluble sugar, cellulase activity, catalase (CAT) activity, and starch. Detailed determination protocols for all indicators are presented in Supplementary Tables S1 and S2 [25].

### 2.5. DNA Extraction, Sequencing, and Data Processing

Total genomic DNA was extracted from 0.2 g rhizosphere soil samples using the Mag-Bind^®^ Soil DNA Kit (Omega Bio-tek, Norcross, GA, USA). DNA integrity and purity were verified via 1% agarose gel electrophoresis, and DNA concentration was quantified with a NanoDrop One to guarantee sample quality. For PE library construction, genomic DNA was sheared to an average fragment size of ~350 bp using a Covaris M220 ultrasonicator (Gene Company Limited, Hong Kong, China). Bridge PCR amplification produced DNA clusters for loading onto the Illumina cBot. Paired-end sequencing was conducted on the Illumina NovaSeq platform with the NovaSeq 6000 S4 Reagent Kit v1.5 (300 cycles) at Majorbio Bio-Pharm Technology Co., Ltd. (Shanghai, China). Raw paired-end reads were quality-filtered with fastp (https://github.com/OpenGene/fastp, version 0.20.0, accessed on 17 January 2024). Reads with low quality (length < 50 bp, Q-score < 20, or ambiguous N bases) were discarded to retain high-quality clean reads ≥ 300 bp. Clean reads were assembled, followed by open reading frame (ORF) prediction. Assembled contigs were subjected to gene prediction and functional annotation. All raw metagenomic datasets generated in this study have been deposited in the NCBI Sequence Read Archive under accession number PRJNA1303850 (https://www.ncbi.nlm.nih.gov/, accessed on 11 April 2024).

### 2.6. Gene Prediction, Taxonomy, and Functional Annotation

Open reading frames (ORFs) were predicted from the clusters from assembled contigs using Prodigal (https://github.com/hyattpd/Prodigal, version 2.6.3, accessed on 28 January 2024). Genes with a nucleic acid length of ≥100 bp were selected and translated into amino acid sequences. The gene sequences predicted from all samples were clustered using CD-HIT software (https://docs.icer.msu.edu/available_software/detail/CD-HIT/, version 4.8.1, accessed on 30 January 2024). Predicted genes with nucleotide sequences of at least 100 bp were retained and translated into amino acid sequences. Gene sequences from all samples were clustered using CD-HIT, with both the sequence-identity and coverage thresholds set to ≥90%. The longest sequence in each cluster was selected as the representative sequence to construct a non-redundant gene catalogue. Before removing redundant sequences, 7,739,206 genes were identified across all samples, with a total sequence length of 3,059,089,209 bp and an average length of 395.27 bp. After clustering, the non-redundant gene catalogue contained 2,938,877 genes, with a total sequence length of 1,290,434,490 bp and an average length of 439.09 bp. SOAPaligner (https://anaconda.org/channels/bioconda/packages/soapaligner/, version 2.21, accessed on 10 February 2024) was used to map high-quality clean reads from each sample to the non-redundant gene catalogue for gene-abundance quantification. DIAMOND (http://ab.inf.uni-tuebingen.de/software/diamond/, version 2.1.8, accessed on 16 February 2024) was used in BLASTp mode to align the non-redundant gene sequences against the NR database. Taxonomic information retrieved from the NR database was used for taxonomic annotation. The abundance of each taxon was calculated by summing the abundances of its matched genes, and taxon-level abundance matrices were generated across samples for subsequent analyses.

Carbon-cycling genes were identified based on KEGG Orthology (KO) identifiers associated with carbon decomposition and fixation and were classified into two functional categories: carbon-decomposition genes and carbon-fixation genes. The carbon-decomposition gene set included genes involved in the degradation of starch, cellulose, hemicellulose, lignin, pectin, chitin, and aromatic compounds. The carbon-fixation gene set included genes associated with major autotrophic carbon-fixation pathways, including the Dicarboxylate–4-hydroxybutyrate cycle, Arnon–Buchanan cycle, 3-hydroxypropionate bicycle, 4-hydroxybutyrate cycle, Calvin cycle, Incomplete reductive citrate cycle, and Wood–Ljungdahl pathway [26,27,28,29]. The key genes and pathways are summarized in Supplementary Tables S3 and S4. Functional genes were taxonomically classified against the NR database. The contribution of a microbial taxon to carbon decomposition or carbon fixation was defined as the sum of the abundances of the corresponding functional genes in that taxon divided by the total abundances of the corresponding functional genes across all microbial taxa involved in the respective function [30].

### 2.7. Statistical Analysis

All statistical analyses and visualizations in this study were performed using R version 4.4.1. The soil physicochemical and plant nutrient data passed the Shapiro–Wilk normality test and Levene’s test for homogeneity of variance. Therefore, independent-sample *t*-tests were used to compare differences in the measured variables between the two restoration strategies, with *p* < 0.05 considered statistically significant. Alpha diversity was calculated using the vegan package, and Venn diagrams were generated using the VennDiagram package. Differences in microbial community composition between the two restoration strategies were assessed using PERMANOVA with 999 permutations, and ANOSIM was performed as a complementary analysis. The abundances of carbon-cycling functional genes were normalized using the Reads Per Kilobase of transcript per Million mapped reads (RPKM) method. To compare the relative abundances of carbon-cycling functional genes in the rhizosphere soils of different plant species, one-way analysis of variance (ANOVA) followed by Tukey’s HSD post hoc multiple-comparison test was performed. The relative abundances of carbon-cycling functional genes were visualized using bubble charts. The Wilcoxon rank-sum test was used to identify microbial families and carbon-cycling functional genes that differed significantly in abundance between the two restoration strategies. Spearman correlation analysis and Mantel tests were performed using the vegan and linkET packages to investigate the relationships between soil environmental factors and the abundances of carbon-cycling functional genes. Correlation matrix heatmaps were generated using the ggcorrplot package.

## 3. Results

### 3.1. Differences in Soil Physicochemical Properties Under Different Restoration Strategies

**Figure 1:**
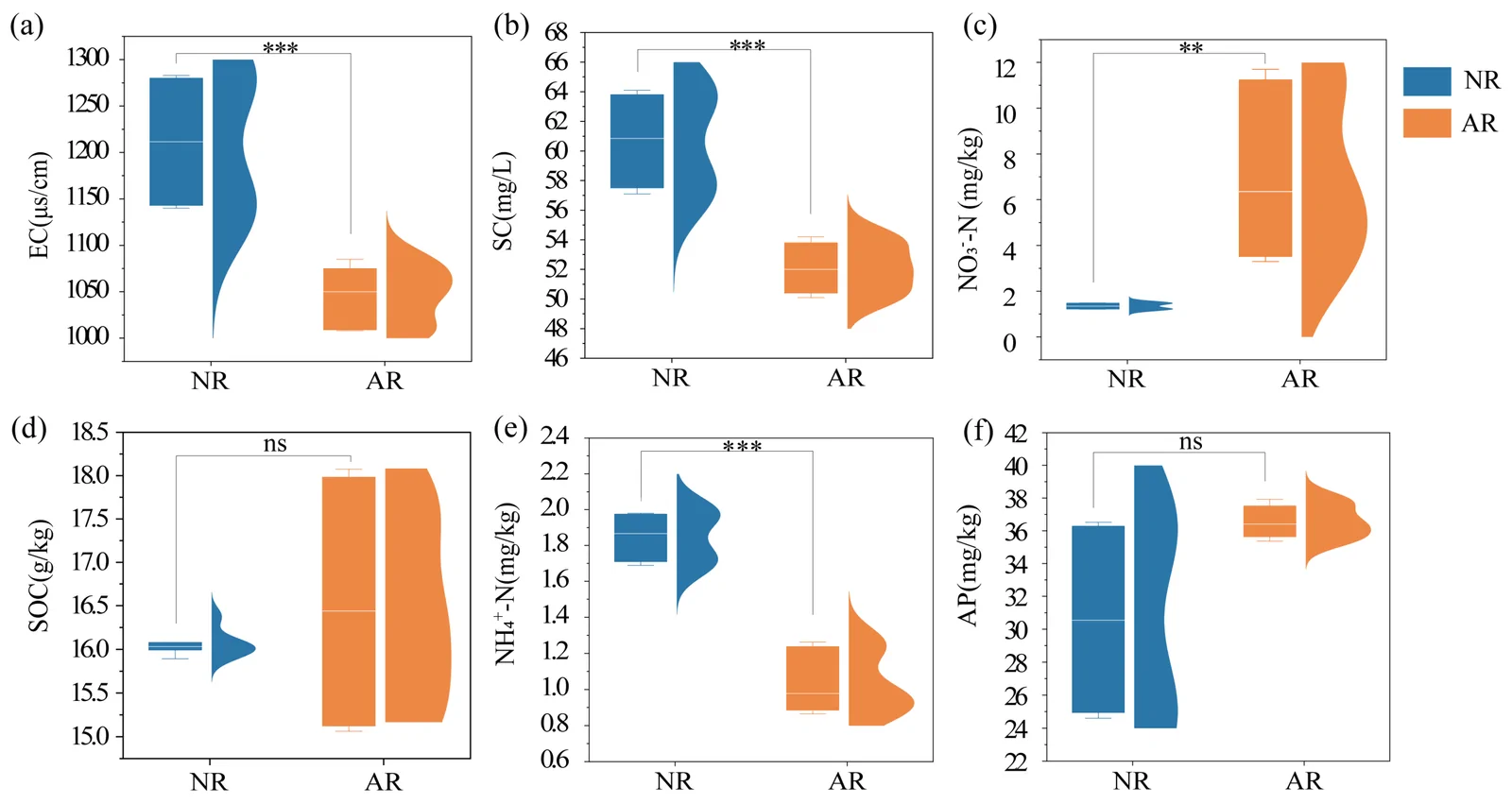
Physicochemical properties of soil under natural recovery (NR) and artificial restoration (AR). (**a**) EC, conductivity; (**b**) SC, salt content; (**c**) NO_3_^−^-N, nitrate nitrogen; (**d**) SOC, soil organic carbon; (**e**) NH_4_^+^-N, ammonium nitrogen; (**f**) AP, available phosphorus. Blue represents NR, and orange represents AR. ns indicates no significant difference; **, and *** indicate significant differences between NR and AR at *p* < 0.01, and *p* < 0.001, respectively.

Soil physicochemical properties differed between the two restoration strategies. Compared with natural recovery (NR), artificial restoration (AR) significantly reduced soil electrical conductivity (EC) and salt content (SC) by 13.63% and 13.70%, respectively (*p* < 0.05; Figure 1a,b). In contrast, nitrate nitrogen (NO_3_^−^-N,) increased significantly by 37.09% under artificial restoration (*p* < 0.01; Figure 1c). Soil organic carbon (SOC) and available phosphorus (AP) also tended to be higher under artificial restoration, increasing by 2.65% and 16.44%, respectively (Figure 1d,f). Conversely, ammonium nitrogen (NH_4_^+^-N) was 44.14% lower under artificial restoration than under natural recovery (Figure 1e).

Soil enzyme activities also differed between the two restoration strategies (Table S5). Phosphatase activity increased by 23.10% under AR relative to NR, whereas urease activity decreased by 5.13%. Among the plant nutrients, total plant tissue total nitrogen (PTN) was significantly higher under AR, increasing by 44.91% relative to natural recovery (*p* < 0.05). Plant starch content also increased by 7.34% under AR. In contrast, plant tissue organic carbon (POC) was 11.10% lower under AR than under NR (Table S6).

### 3.2. Differences in Rhizosphere Soil Microbial Communities Under Different Restoration Strategies

**Figure 2:**
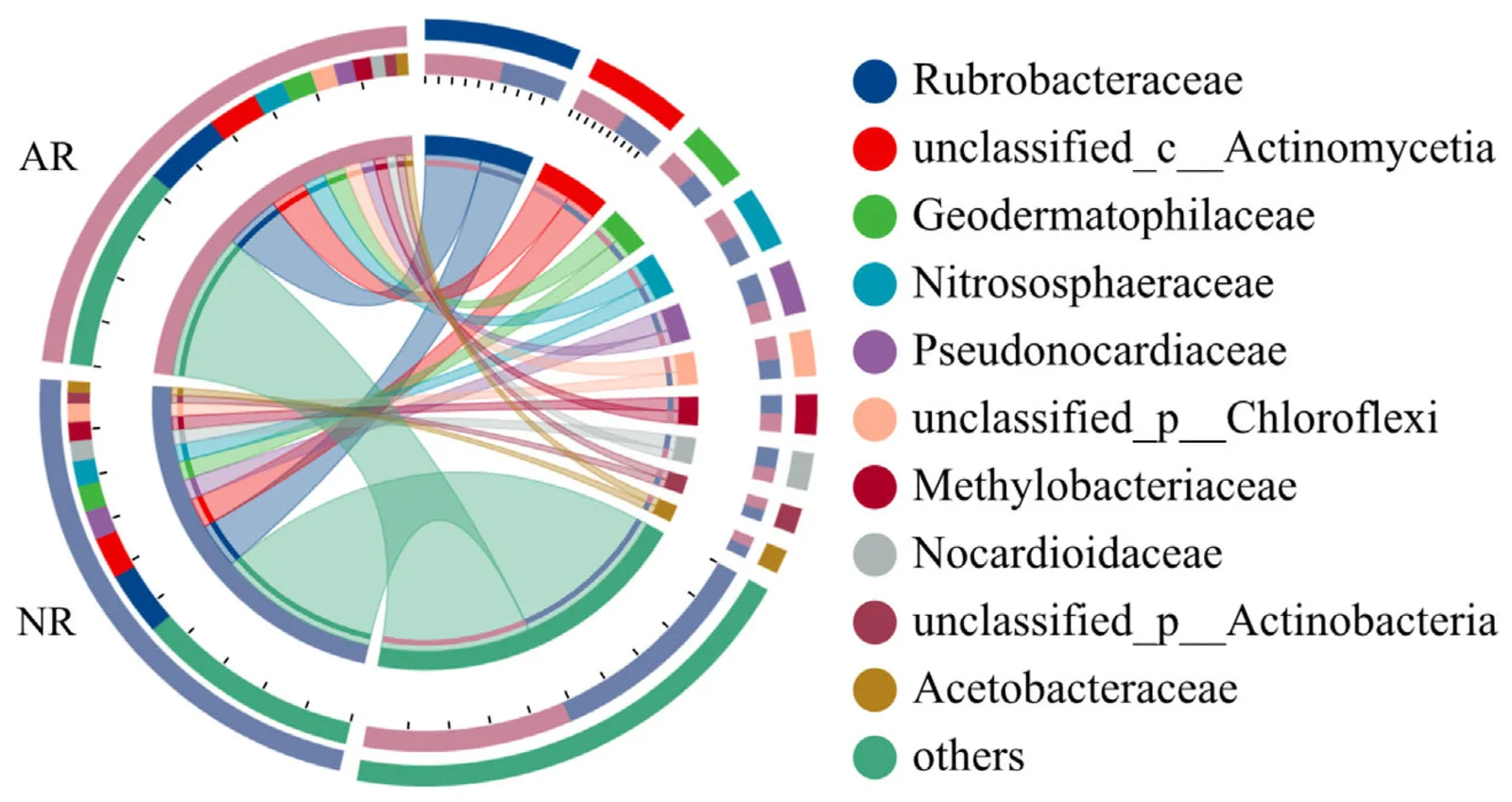
Circos plot of microbial communities at the family taxonomic level. NR, natural recovery; AR, artificial restoration. Black tick marks around the circle represent abundance scale ticks, with each interval representing 10% relative abundance.

At the domain level, microbial community composition did not differ significantly between the two restoration strategies (Table S7). At the phylum classification level, NR and AR treatments shared 218 OTUs, which accounted for 94.78% of the total detected OTUs across all samples. In contrast, four OTUs were unique to NR (1.74% of total OTUs), while eight OTUs were exclusive to AR (3.48% of total OTUs). Across all samples, Actinobacteria were the dominant phylum, followed by Proteobacteria, Chloroflexi, Thaumarchaeota, Acidobacteria, and Gemmatimonadetes. Together, these dominant phyla accounted for 89.98–93.63% of all detected OTUs (Figure S1). At the class level, the relative abundances of Chloroflexia, Thermomicrobia, and Gemmatimonadetes were higher under AR than under NR (Figure S2). At the family level, Rubrobacteraceae, unclassified_c__Actinomycetia, unclassified_p__Chloroflexi, unclassified_p__Actinobacteria, and unclassified_o__Solirubrobacterales were significantly enriched under AR (*p* < 0.05; Figure 2 and Figure S3).

**Figure 3:**
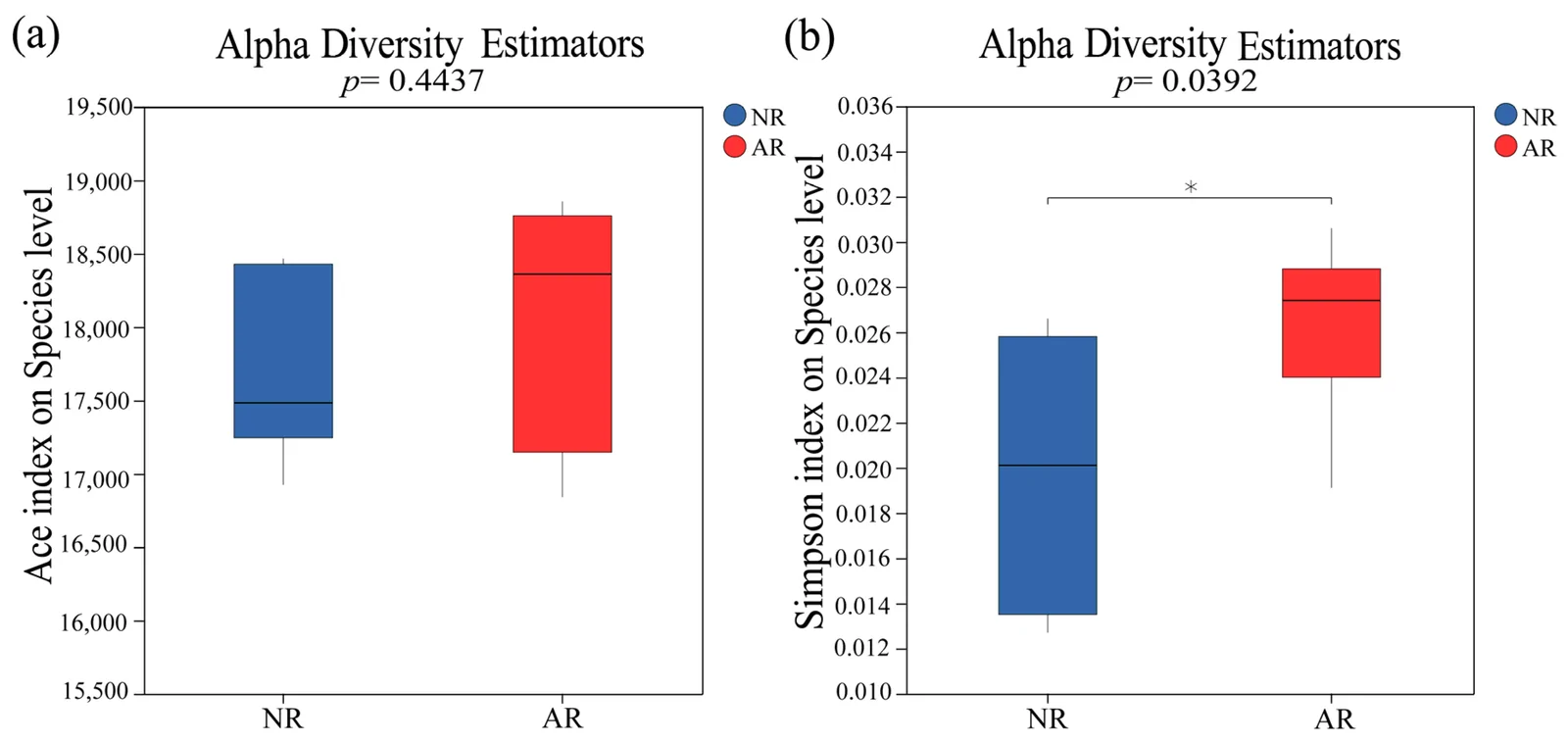
α-diversity of NR and AR. (**a**) ACE index and (**b**) Simpson index. NR, natural recovery; AR, artificial restoration. * indicates significant differences at *p* < 0.05.

The α-diversity indices further revealed distinct responses of microbial richness and diversity to restoration strategies. The ACE richness index increased slightly under AR compared with NR (Figure 3a), whereas the Simpson diversity index was significantly higher under AR (*p* < 0.05; Figure 3b). Significant differences in microbial communities at the family taxonomic level were observed between the two restoration strategies, indicating that restoration strategy is a significant, but not the sole, driver of community composition (Table S8). Principal coordinate analysis showed that the microbial community associated with *L. frutescens* under natural recovery was positioned close to the communities associated with plant species under AR, whereas the community associated with *B. scoparia* was more clearly separated from those associated with the other plant species (Figure S4). This suggests that plant identity may also influence rhizosphere microbial community composition within each restoration strategy. 

### 3.3. Differences in Carbon Cycling Under Different Restoration Strategies

#### 3.3.1. The Abundances of Carbon-Decomposition-Related Genes

**Figure 4:**
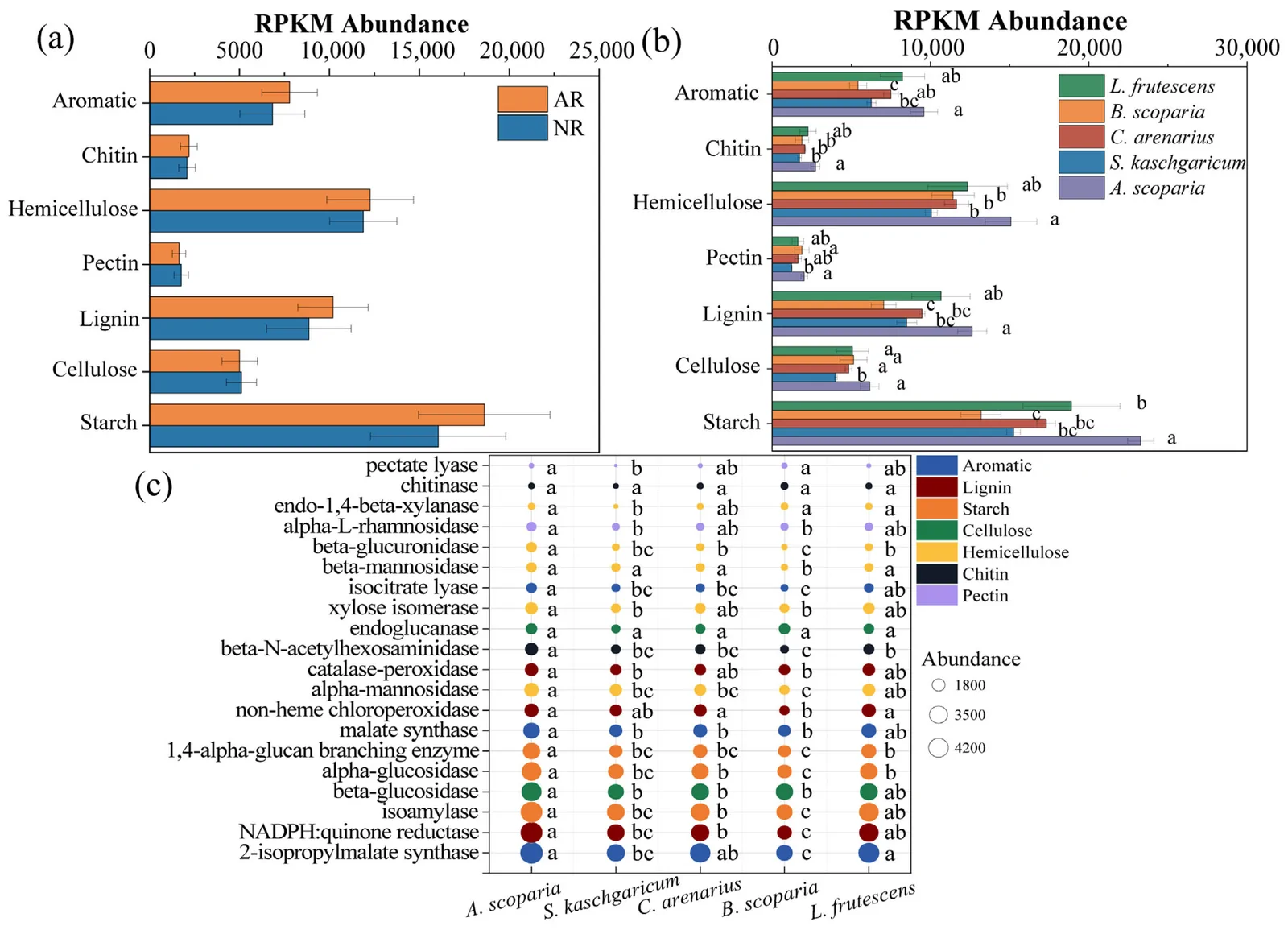
Soil carbon decomposition function. (**a**) Gene RPKM abundances of Starch, Cellulose, Hemicellulose, Lignin, Chitin, Pectin, and Aromatics under different restoration strategies. (**b**) Gene RPKM abundances of Aromatics, Chitin, Hemicellulose, Pectin, Lignin, Cellulose, and Starch under different plant species. (**c**) Gene RPKM abundances within each taxonomic group. NR, natural recovery; AR, artificial restoration. *A. scoparia*, *Artemisia scoparia*; *S. kaschgaricum*, *Seriphidium kaschgaricum*; *C. arenarius*, *Ceratocarpus arenarius*; *B. scoparia*, *Bassia scoparia*; *L. frutescens*, *Leucophyllum frutescens*. Different lowercase letters indicate statistically significant differences among plant species (*p* < 0.05).

Non-metric multidimensional scaling (NMDS) analysis revealed significant differences in the profiles of carbon-decomposition-related genes between the two restoration strategies (Figure S5a). Compared with NR, AR increased the relative abundances of several functional genes involved in carbon decomposition, particularly those associated with the degradation of aromatic compounds, lignin, and starch (Figure 4a). The abundances of carbon-decomposition-related genes also differed significantly among the rhizosphere soils of the five dominant plant species (Figure 4b). Further analysis of the core functional genes involved in carbon decomposition showed that the genes encoding 2-isopropylmalate synthase and malate synthase were key genes associated with aromatic-compound degradation. The major genes involved in lignin degradation encoded NADPH:quinone reductase and non-haem chloroperoxidase, whereas starch degradation was primarily associated with genes encoding isoamylase, alpha-glucosidase, and 1,4-alpha-glucan branching enzyme. Hemicellulose degradation was mainly associated with genes encoding alpha-mannosidase, beta-mannosidase, and beta-glucuronidase, while the gene encoding beta-N-acetylhexosaminidase was identified as a marker gene for chitin degradation (Figure 4c). Overall, the abundances of the above-mentioned genes in the rhizosphere soil of *B. scoparia* were generally lower than those in the rhizosphere soils of *A. scoparia*, *S. kaschgaricum*, and *C. arenarius*. In contrast, the abundances of these genes in the rhizosphere soil of *L. frutescens* under NR were comparable to those observed in the rhizosphere soils of *A. scoparia*, *S. kaschgaricum*, and *C. arenarius* under AR (Figure 4b). In contrast, the abundances of these genes in the rhizosphere soil of *L. frutescens* under NR were comparable to those observed in the rhizosphere soils of *A. scoparia*, *S. kaschgaricum*, and *C. arenarius* under AR (Figure 4b).

#### 3.3.2. Microbial Families Associated with Soil Carbon-Decomposition-Related Genes

**Figure 5:**
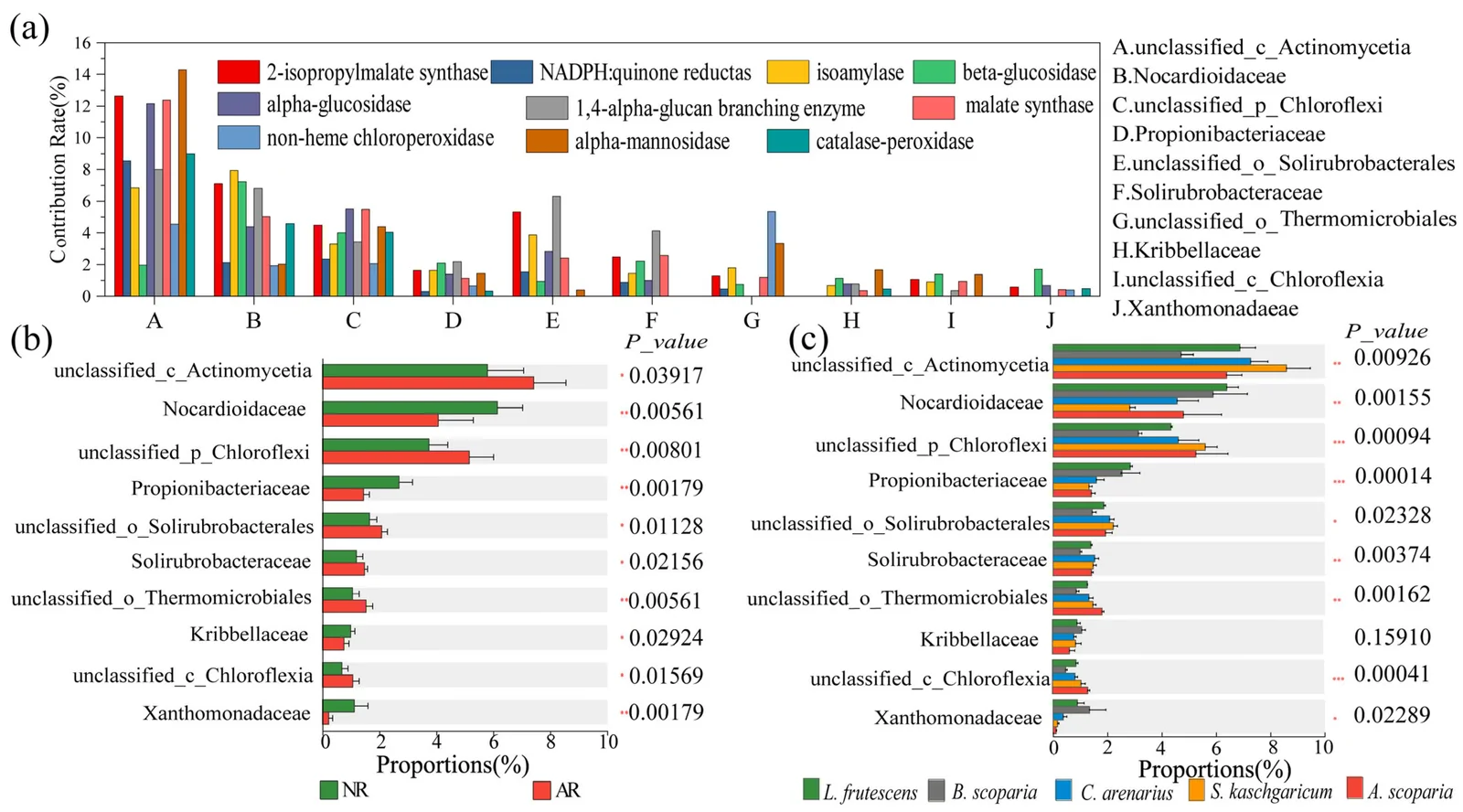
Contributions of microbial families to carbon decomposition genes and their abundance differences under different restoration strategies. (**a**) Contributions of dominant microbial families to differential carbon decomposition genes under natural and artificial restoration; (**b**) Abundance variations in key microbial families between NR and AR; (**c**) Abundance differences in key microbial families among different plant species. NR, natural recovery; AR, artificial restoration. *, **, and *** indicate significant differences at *p* < 0.05, *p* < 0.01, and *p* < 0.001, respectively.

A total of 467 microbial families were annotated in the carbon-decomposition-related gene set, 65 of which differed significantly between the two restoration strategies (*p* < 0.05). The top ten microbial families with the greatest differences in the composition of carbon decomposition functional genes under different restoration strategies are as follows: unclassified_c__Actinomycetia, Nocardioidaceae, unclassified_p__Chloroflexi, Propionibacteriaceae, unclassified_o__Solirubrobacterales, Solirubrobacteraceae, unclassified_o__Thermomicrobiales, Kribbellaceae, unclassified_c__Chloroflexia, and Xanthomonadaceae. Contribution analysis of the top 50 microbial families to functional gene pools revealed that unclassified_c__Actinomycetia, Nocardioidaceae, unclassified_p__Chloroflexi and Propionibacteriaceae dominated the gene pools of the ten most abundant enzyme genes involved in carbon decomposition (Figure 5a). Members of the order Solirubrobacterales dominated the abundance of eight enzyme genes, excluding *non-haem chloroperoxidase* and *catalase-peroxidase*.

Differential analysis further demonstrated that six microbial groups, unclassified_c__Actinomycetia, unclassified_p__Chloroflexi, unclassified_o__Solirubrobacterales, Solirubrobacteraceae, unclassified_o__Thermomicrobiales and unclassified_c__Chloroflexia, were significantly enriched in the rhizosphere soils of the four plant species except *B. scoparia* (Figure 5c). The relative abundances of these six microbial families were higher under AR than under NR (Figure 5b). These microbial families may be important contributors to the observed differences in carbon-decomposition-related gene genetic potential.

#### 3.3.3. The Abundances of Carbon-Fixation-Related Genes

**Figure 6:**
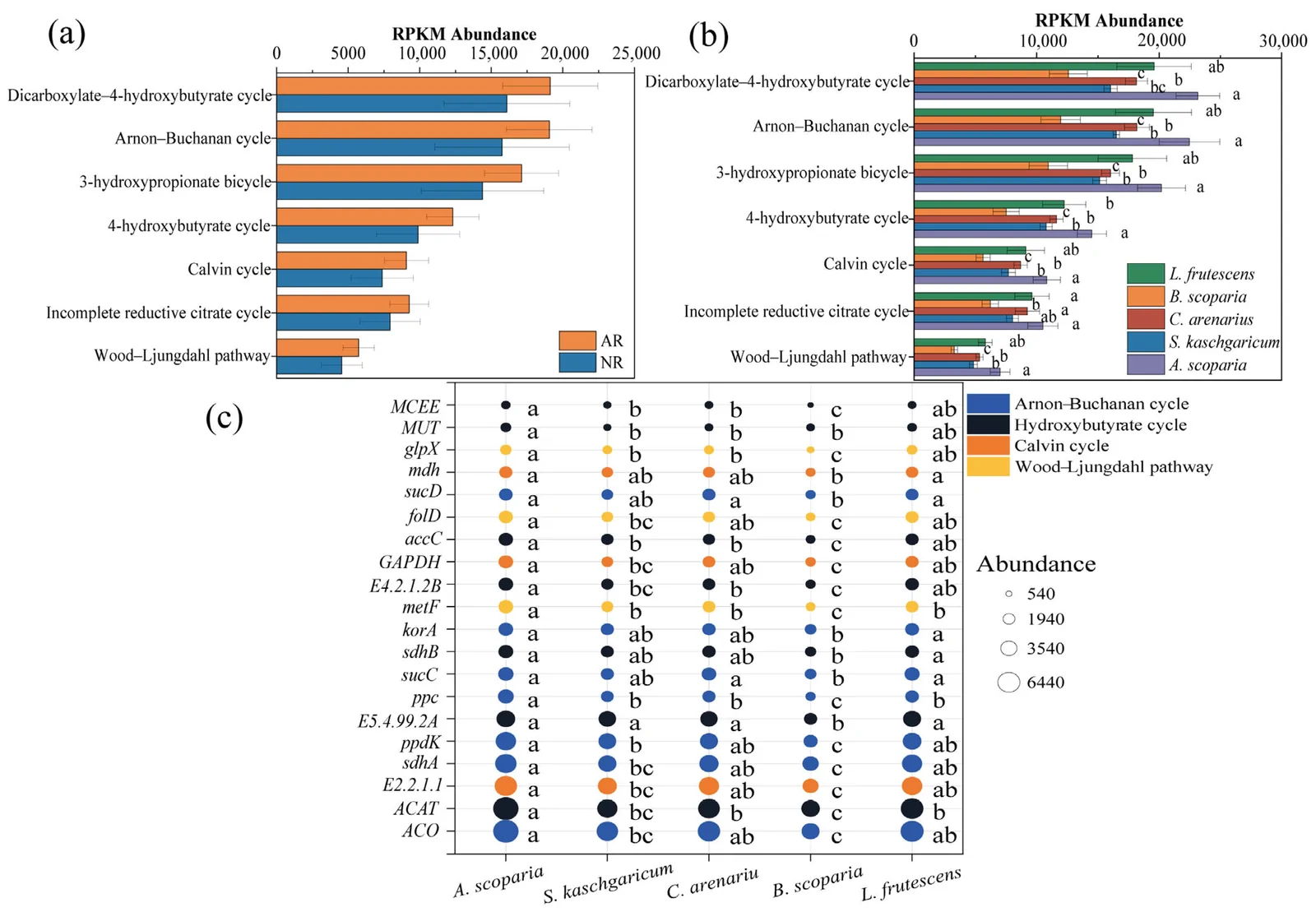
Soil carbon fixation function. (**a**) RPKM abundance of carbon fixation pathway genes under different restoration strategies. (**b**) RPKM abundance of carbon fixation pathway genes among different plant species. (**c**) RPKM abundance of genes across taxonomic groups. NR, natural recovery; AR, artificial restoration. *A. scoparia*, *Artemisia scoparia*; *S. kaschgaricum*, *Seriphidium kaschgaricum*; *C. arenarius*, *Ceratocarpus arenarius*; *B. scoparia*, *Bassia scoparia*; *L. frutescens*, *Leucophyllum frutescens*. Different lowercase letters indicate statistically significant differences among plant species (*p* < 0.05).

NMDS analysis revealed significant differences in the profiles of carbon-fixation-related genes between the two restoration strategies (Figure S5b). The gene abundances of carbon-fixation-related pathways were higher under AR than under NR (Figure 6a). Among the five plant species, carbon fixation pathway abundances in the rhizosphere soils of *B. scoparia* were significantly lower than those in the soils of *A. scoparia*, *S. kaschgaricum*, and *C. arenarius*. In contrast, *L. frutescens* displayed an abundance pattern similar to that observed for these three species (Figure 6b). Further analysis of genes involved in carbon fixation pathways (Figure 6c) showed that several carbon-fixation-related genes were significantly enriched (*p* < 0.05) in the rhizosphere soils of the four plant species other than *B. scoparia*. These genes included *pyruvate orthophosphate dikinase* (*ppdK*), *methylmalonyl-CoA mutase* (*E5.4.99.2A*), *phosphoenolpyruvate carboxylase* (*ppc*), *5,10-methylenetetrahydrofolate reductase* (*metF*), *acetyl-CoA carboxylase biotin carboxylase subunit* (*accC*), *methylenetetrahydrofolate dehydrogenase/cyclohydrolase* (*folD*), *succinyl-CoA synthetase alpha subunit* (*sucD*), *fructose-1,6-bisphosphatase II* (*glpX*), and *methylmalonyl-CoA epimerase* (*MCEE*). The results reflect the genetic potential of microbial carbon fixation.

#### 3.3.4. Microbial Families Associated with Soil Carbon-Fixation-Related Genes

**Figure 7:**
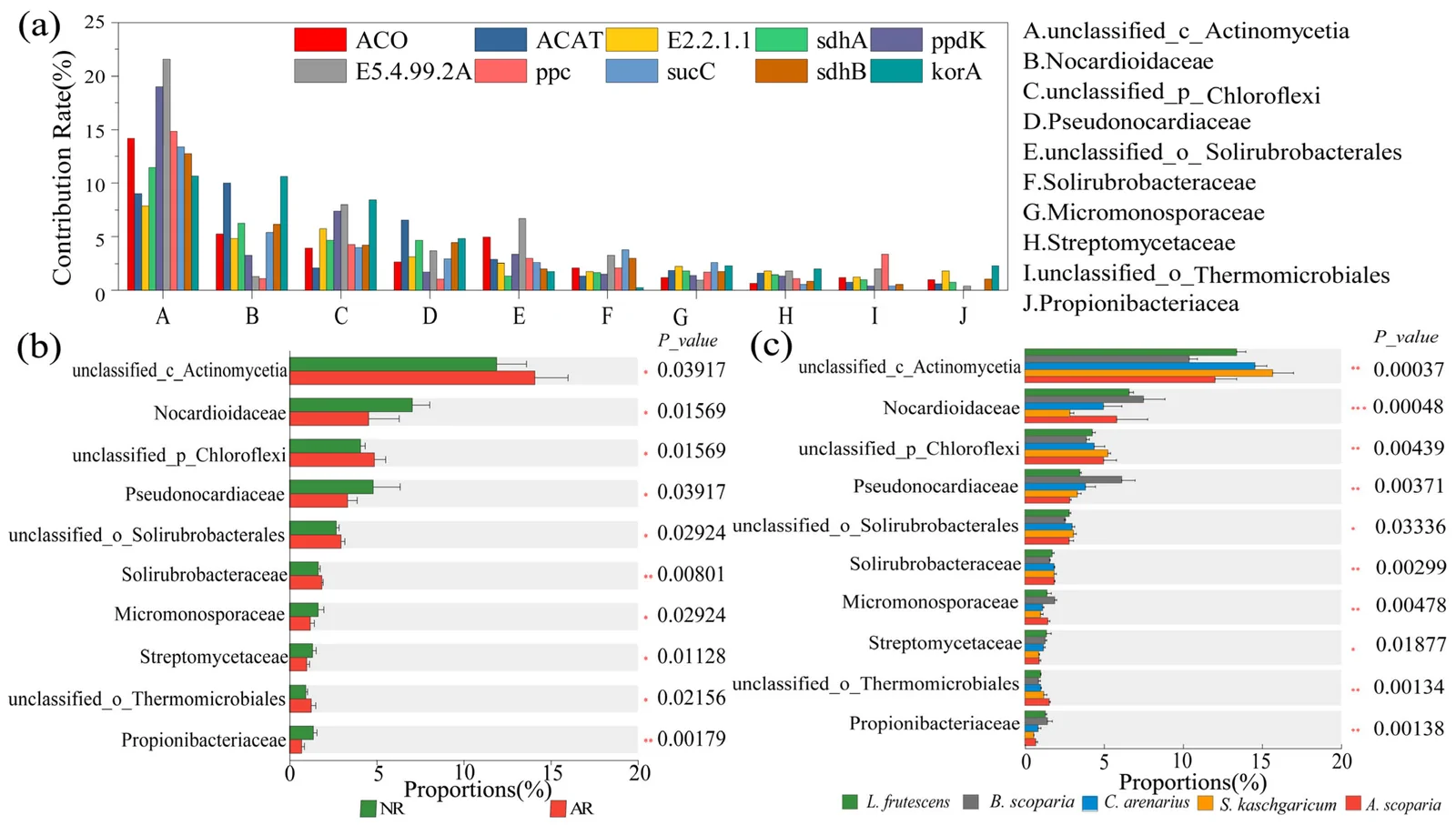
Contributions of microbial families to carbon-fixation-related genes and their abundance differences under different restoration strategies. (**a**) Contributions of microbial families to differential carbon-fixation-related genes under different restoration strategies; (**b**) Family-level abundance differences in key microbial taxa between different restoration strategies; (**c**) Family-level abundance differences in key microbial taxa among different plant species. NR, natural recovery; AR, artificial restoration. *, **, and *** indicate significant differences at *p* < 0.05, *p* < 0.01, and *p* < 0.001, respectively.

A total of 445 microbial families were annotated in the carbon-fixation-related gene set, 51 of which differed significantly between the two restoration strategies (*p* < 0.05). Among the top 50 most abundant families, ten displayed distinct contributions to carbon fixation gene profiles across restoration strategies: unclassified_c__Actinomycetia, Nocardioidaceae, unclassified_p__Chloroflexi, Pseudonocardiaceae, unclassified_o__Solirubrobacterales, Solirubrobacteraceae, Micromonosporaceae, Streptomycetaceae, unclassified_o__Thermomicrobiales, and Propionibacteriaceae. Taxonomic contribution analysis for carbon fixation genes revealed that all ten families were primary contributors to *aconitate hydratase (ACO)*, *acetyl-CoA acetyltransferase (ACAT)*, *transketolase (EC 2.2.1.1)*, *succinate dehydrogenase flavoprotein subunit (sdhA)*, *methylmalonyl-CoA mutase subunit (E5.4.99.2A)*, and *succinate dehydrogenase iron–sulfur subunit (sdhB)* (Figure 7a; Table S9). In addition, the relative abundances of *pyruvate, orthophosphate dikinase (ppdK)*, *phosphoenolpyruvate carboxylase (ppc)*, and *succinyl-CoA synthetase beta subunit (sucC)* were mainly derived from unclassified_c__Actinomycetia, Nocardioidaceae, unclassified_p__Chloroflexi, Pseudonocardiaceae, unclassified_o__Solirubrobacterales, Solirubrobacteraceae, Micromonosporaceae, Streptomycetaceae, and unclassified_o__Thermomicrobiales. The *2-oxoglutarate: ferredoxin oxidoreductase alpha subunit (korA)* gene abundance was mainly linked to the above nine microbial families, excluding unclassified_o__Thermomicrobiales. Differential abundance analysis demonstrated that unclassified_c__Actinomycetia, unclassified_p__Chloroflexi, unclassified_o__Solirubrobacterales, Solirubrobacteraceae, and unclassified_o__Thermomicrobiales were significantly enriched in soils under AR relative to NR (*p* < 0.05; Figure 7b). Their relative abundances also varied markedly among the five plant species; the rhizosphere of *L. frutescens* showed abundance profiles comparable to those of *A. scoparia*, *S. kaschgaricum*, and *C. arenarius* (Figure 7c). Collectively, these microbial families drove the observed divergence in carbon-fixation-related genetic potential.

### 3.4. Relationships Between Environmental Factors and Carbon Cycle-Related Genes

**Figure 8:**
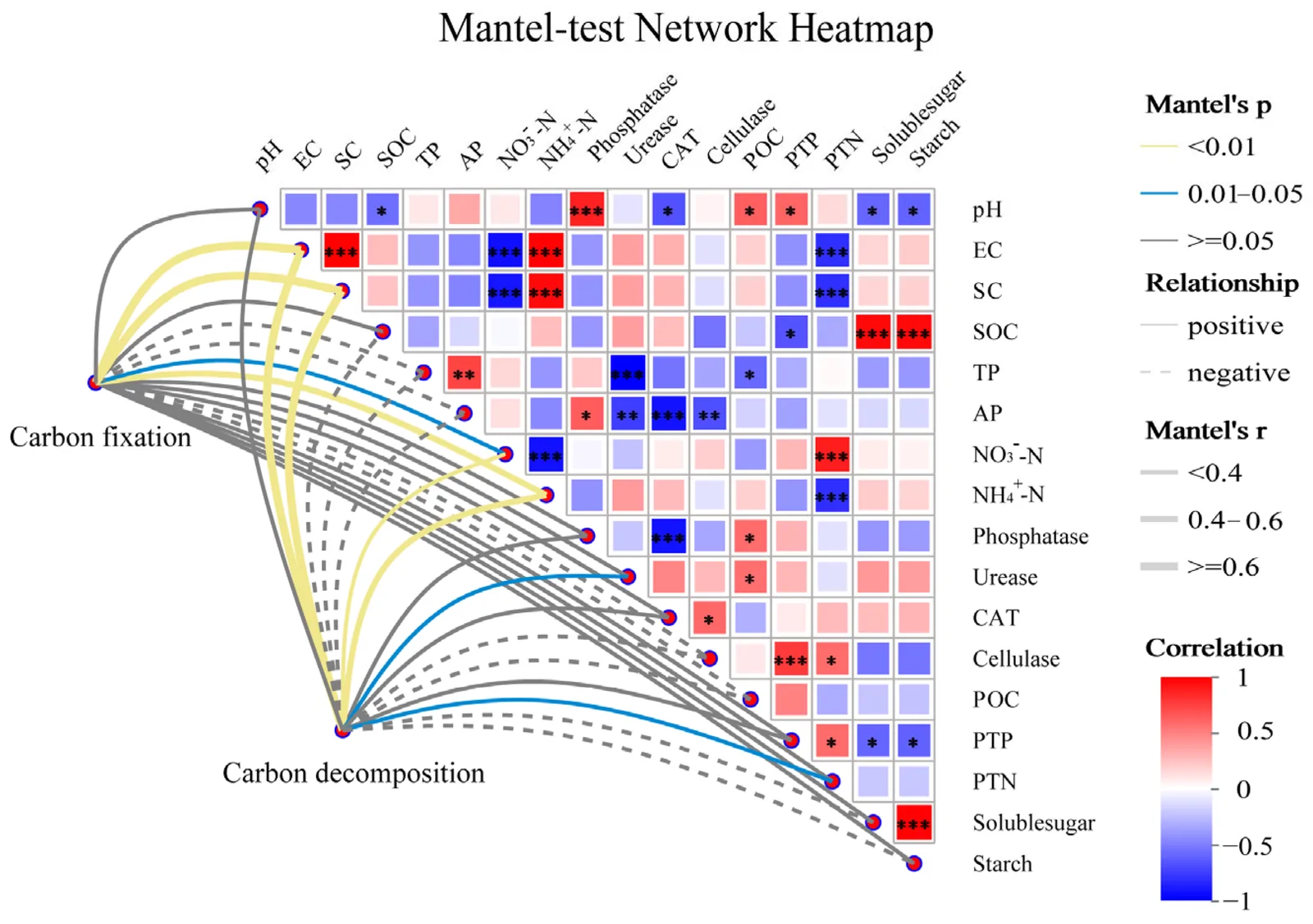
Mantel analysis revealing the correlations between environmental factors and carbon cycling-related genes. Redder color denotes stronger positive correlation, while bluer color indicates stronger negative correlation. Statistical significance was defined as follows: * *p* < 0.05 was considered significant, ** *p* < 0.01 was considered highly significant, and *** *p* < 0.001 was considered extremely significant. Soil indicators: pH, soil pH; EC, electrical conductivity; SC, salt content; SOC, soil organic carbon; TP, total phosphorus; AP, available phosphorus; NO_3_^−^-N, nitrate nitrogen; NH_4_^+^-N, ammonium nitrogen; Phosphatase, phosphatase activity; Urease, urease activity. Plant-tissue variables: CAT, catalase activity; Cellulase, cellulase activity; POC, plant tissue organic carbon; PTP, plant tissue total phosphorus; PTN, plant tissue total nitrogen; Soluble sugar, plant soluble sugar; Starch, plant starch.

Mantel analysis (Figure 8) showed that genes related to carbon decomposition were significantly positively correlated with environmental factors such as EC, SC, NO_3_^−^-N, NH_4_^+^-N, urease, and PTN, and negatively correlated with SOC, TP, AP, cellulase, POC, plant soluble sugar, and starch. Carbon-fixation-related genes showed significant positive correlations with EC, SC, NO_3_^−^-N, and NH_4_^+^-N (*p* < 0.05).

## 4. Discussions

### 4.1. Soil Characteristics in Response to Different Restoration Strategies

Improvements in soil physicochemical properties serve as core indicators for evaluating vegetation restoration effectiveness in mining areas [31,32]. In this study, artificial restoration significantly reduced soil salinity and electrical conductivity in the mining area, which was consistent with previous findings demonstrating that artificial vegetation planting alleviated soil salinization in mining ecosystems [33]. These improvements may stem from the higher vegetation density and surface coverage under artificial restoration, which accelerated vegetation layer formation, reduced surface soil evaporation, and inhibited topsoil salt accumulation [34]. Furthermore, increased vegetation coverage enhanced root metabolic activity and mitigated soil salt stress via ion adsorption and root exudate secretion [35]. Increases in soil organic carbon, nitrate nitrogen, available phosphorus, total phosphorus, and plant total nitrogen content under artificial restoration reflected enhanced soil nitrogen and phosphorus supply capacity, which were closely related to the dominant plant species in this restoration mode. Such differences in soil physicochemical traits can be explained by the fact that higher plant diversity under artificial restoration optimized the rhizosphere microecosystem and improved soil physicochemical properties through complex root–soil interactions [36].

### 4.2. Microbial Community Responses to Different Restoration Strategies

Soil microbial communities play a vital role in biogeochemical cycling and plant growth, and microbial diversity is widely used as an indicator for evaluating the recovery of mining ecosystems [37]. In this study, microbial α-diversity and OTU-level community dissimilarity were higher under artificial restoration than under natural recovery. Differential responses of the ACE and Simpson indices revealed that artificial restoration reshaped microbial community structure primarily by altering the relative abundance of microbial taxa, rather than substantially enhancing species richness. The significant increase in the Simpson index further indicated that artificial restoration weakened the competitive dominance of specific microbial taxa and increased community evenness. This pattern may be associated with early management practices or plant assemblages under different restoration strategies [38,39]. Actinobacteria, Proteobacteria, and Acidobacteria were widely detected across all samples. These phyla possessed wide environmental adaptability, which was consistent with the findings of previous studies on microbial communities in mining areas [40]. However, our sampling site is representative of typical arid coal mining subsidence terrain, so our results only apply to arid coal mining subsidence zones with similar climate, restoration history, and vegetation communities. These findings should be used with caution when extrapolated to other mining areas with distinct environmental conditions or degradation processes.

Although PERMANOVA demonstrated that restoration strategy significantly altered microbial community structure at the family level (R^2^ ≈ 0.237, *p* = 0.028), its low explanatory power revealed that restoration only accounted for approximately one-quarter of the total microbial community variation. In addition, the PCoA results demonstrated that the rhizosphere microbial community of *L. frutescens* under natural recovery was analogous to those of multiple plant species under artificial restoration, while *B. scoparia* harbored a distinctly different microbial community. This indicates that the restoration strategy was a significant but partial driver of microbial community assembly, rather than the sole determinant. Analyses at the plant species level further revealed that microbial community structure may be co-determined by restoration regimes and plant species types [41]. Notably, restoration strategy and vegetation composition were partly coupled in this field study. Therefore, the observed variations in rhizosphere microbial communities and carbon-cycling-related gene profiles reflected the comprehensive effects of integrated restoration methods, rather than the independent influence of restoration strategy alone. Consequently, plant species identity should be fully considered in future vegetation restoration studies. Importantly, this study highlights the ecological significance of rational plant species selection. Naturally occurring species such as *L. frutescens* have great potential for guiding the vegetation reconstruction of degraded mining ecosystems.

### 4.3. Shifts in Carbon Cycling Gene Profiles and Corresponding Microbial Communities Across Restoration Strategies

The presence of carbon decomposition genes in rhizosphere microorganisms was crucial for the breakdown and utilization of carbon sources in soil, and there is a strong correlation between these genes and their corresponding enzyme activities [27]. In this study, the relative abundances of genes associated with the decomposition of aromatic compounds, lignin, and starch were generally higher under artificial restoration than under natural recovery, suggesting that artificial restoration may only alter the genetic potential for the degradation of certain carbon substrates. Furthermore, the between-group differences in the abundance of carbon-decomposition-related genes may be associated with soil total nitrogen and other nutrients. Meanwhile, changes in vegetation-derived organic matter inputs [42], rhizosphere nutrient conditions and plant species identity under different restoration strategies also acted as important influencing factors [43]. These relationships suggested that rhizosphere environmental conditions were linked to the distribution of carbon-decomposition-related genes. By evaluating the contributions of microbial communities to carbon-decomposition-related genes, we found that Actinomycetia, Solirubrobacterales, Chloroflexi, and Thermomicrobiales were important contributors to several carbon-decomposition-related genes under artificial restoration. For example, the higher relative abundance of isoamylase genes under artificial restoration was mainly associated with Actinomycetia, Chloroflexi, Solirubrobacterales, and Thermomicrobiales [44,45]. Actinomycetes also contributed to genes associated with aromatic compound decomposition, suggesting that they harbored microbial groups with genetic potential for aromatic carbon transformation [46,47,48,49]. The phylum Chloroflexi contains the class Thermomicrobia. Under artificial restoration conditions, elevated abundances of these two microbial groups boosted the activities of carbon-degrading enzymes, which likely facilitated soil organic matter transformation and energy flow within the soil carbon cycle of mining sites [50]. Furthermore, Actinobacteria and Chloroflexi carry genes encoding enzymes for lignin and cellulose catabolism, rendering them key participants in the decomposition of these recalcitrant substrates [51]. These findings indicated that shifts in specific microbial families were associated with differences in the genetic potential of carbon-decomposition-related genes.

Improving the carbon fixation potential of rhizosphere microbial communities may be important for the recovery of mining ecosystems [52]. In this study, the relative abundances of several carbon-fixation-related genes were higher under artificial restoration than under natural recovery, suggesting that artificial restoration was associated with greater microbial genetic potential for carbon fixation [53]. The higher relative abundances of carbon-fixation-related genes under artificial restoration may reflect changes in microbial community composition and rhizosphere environmental conditions. Previous studies have reported that several carbon-fixing microbial groups are affiliated with Actinobacteria, Proteobacteria, Acidobacteria, and Chloroflexi [54,55,56,57]. In the present study, some microbial families within these groups were enriched under artificial restoration and contributed to carbon-fixation-related genes, indicating that they may be important carriers of carbon fixation-related genetic potential [58]. Although the relative abundances of several carbon fixation-associated microbial groups increased under artificial restoration, the overall differentiation in microbial community structure between the two restoration strategies was non-significant. This further indicates that factors including vegetation type, vegetation coverage, restoration duration, soil depth, and climatic conditions may also cause differences in the genetic potential for soil microbial carbon sequestration in mining areas [59,60]. In addition, the salt content of the soil and the nitrogen nutrient content are significantly correlated with the carbon cycle genes, indicating that they may be important environmental factors affecting the carbon cycle [61]. These factors can influence rhizosphere microbial community composition and the distribution of microbial functional genes. In this study, ten microbial families were identified as key taxa driving differences in carbon-fixation-related gene profiles in this study, which provide a reference for identifying microbial groups with genetic potential for carbon sequestration under different restoration strategies. Nevertheless, further validation combining metatranscriptomic analysis, gene expression quantification and carbon flux measurements is required to clarify whether these genes can induce tangible shifts in soil carbon decomposition processes.

## 5. Conclusions

In summary, soils under artificial restoration displayed lower electrical conductivity and salt content relative to natural recovery, accompanied by ameliorated soil environmental conditions. In addition, artificial restoration reshaped rhizosphere microbial community structure and significantly elevated the relative abundances of Actinomycetia, Solirubrobacterales, and Chloroflexi. These microbial taxa were the primary contributors to genes responsible for aromatic compound, lignin, and starch degradation, and were also correlated with multiple functional genes governing microbial carbon fixation pathways. Furthermore, the distinct clustering of rhizosphere microbial communities associated with *L. frutescens* under natural restoration and various plant species under artificial restoration verified that plant traits served as key drivers shaping the assembly of carbon-cycle-related functional microbial communities. This study has several limitations. Although our main comparison centered on soil and microbial properties across distinct restoration modes, we did not conduct quantitative analyses to disentangle individual plant species effects or interactive effects between restoration regime and plant identity. Future research should establish standardized controlled vegetation experiments and expand sampling coverage to multiple mining subsidence sites, so as to further clarify the underlying regulatory mechanisms and improve the generalizability of our findings. Importantly, artificial restoration enriched carbon-cycle-related microbial taxa. This pattern may facilitate the shift in mine soils from carbon sources to carbon sinks and provide a theoretical basis for elevating carbon sequestration potential in degraded mine soils and refining restoration strategies for arid saline-alkali mining ecosystems.

## Data Availability

The original contributions presented in this study are included in the article/Supplementary Materials. Further inquiries can be directed to the corresponding author.

## Supplementary Materials

The following supporting information can be downloaded at: https://www.mdpi.com/article/10.3390/microorganisms14081713/s1. Figure S1: Venn diagram showing shared and unique OTUs at the phylum level. NR, natural recovery; AR, artificial restoration. Figure S2: There were significant differences in microbial classes under different restoration strategies. NR, natural recovery; AR, artificial restoration. Figure S3: Microbial families with significant differences under different restoration strategies. NR, natural recovery; AR, artificial restoration. Figure S4: Classification level of microbial family PCoA. NR, natural recovery; AR, artificial restoration. *A. Scoparia, Artemisia scoparia; S. Kaschgaricum, Seriphidium kaschgaricum; C. Arenarius, Ceratocarpus arenariu; B. Scoparia, Bassia scoparia; L. Frutescens, Leucophylum frutescens.* Figure S5: NMDS analysis on the carbon decomposition genes (a) and carbon-fixation-related genes (b) difference under different restoration strategies. NR, natural recovery; AR, artificial restoration. Table S1: Soil Variables and Detection Methods. Table S2: Plant-tissue Variables and Detection Methods. Table S3: The function, gene and KO of carbon decomposition. Table S4: The function, gene and KO of carbon fixation. Table S5: Differences in soil physical and chemical properties under different restoration strategies. Table S6: Differences in plant-tissue physical and chemical properties under different restoration strategies. Table S7: *p*-values for differences in relative abundances of microbial domains between the two restoration strategies. Table S8: PERMANOVA results (permutational multivariate analysis of variance) comparing microbial community structure at the family level between the two restoration strategies; Table S9: The KO, gene abbreviation and full name of carbon fixation.
