# Supplementary material for: Dominant Effect of Ecological Restoration on Microbial Carbon Cycle in Plant Rhizosphere of Mining Areas
Source: Microorganisms. 2026 Aug 4;14(8):1713. doi: 10.3390/microorganisms14081713 (PMC13515858; doi:10.3390/microorganisms14081713)
Supplement: Supplementary file 1 [file microorganisms-14-01713-s001.zip › microorganisms-4399137-supplementary.pdf]

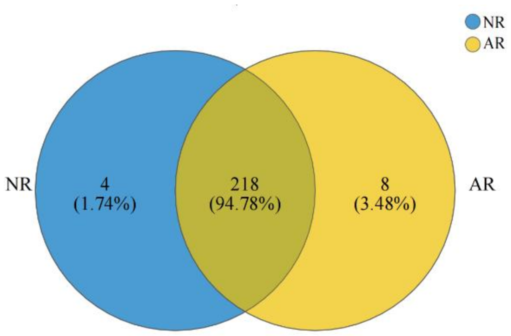

Figure S1. Venn diagram showing shared and unique OTUs at the phylum level. NR, natural recovery; AR, artificial restoration.

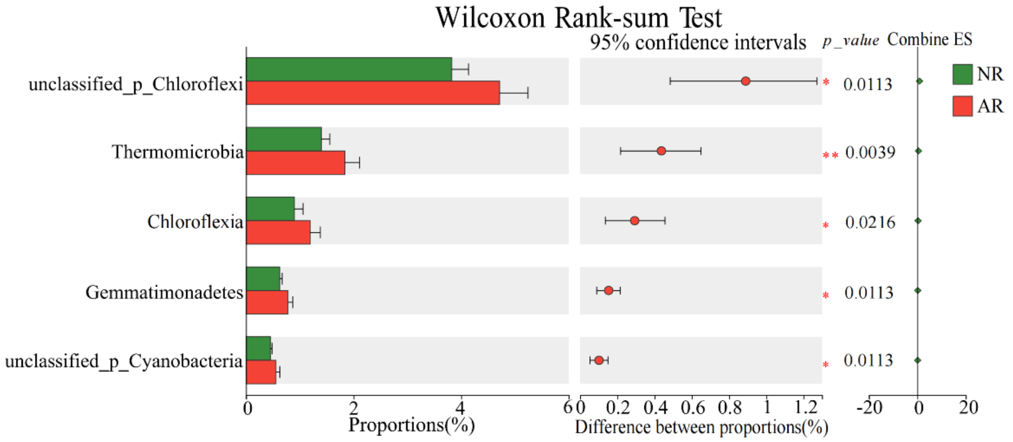

Figure S2. There were significant differences in microbial classes under different restoration strategies. NR, natural recovery; AR, artificial restoration. * and ** indicate significant differences between NR and AR at $p < 0.05$ and $p < 0.01$, respectively.

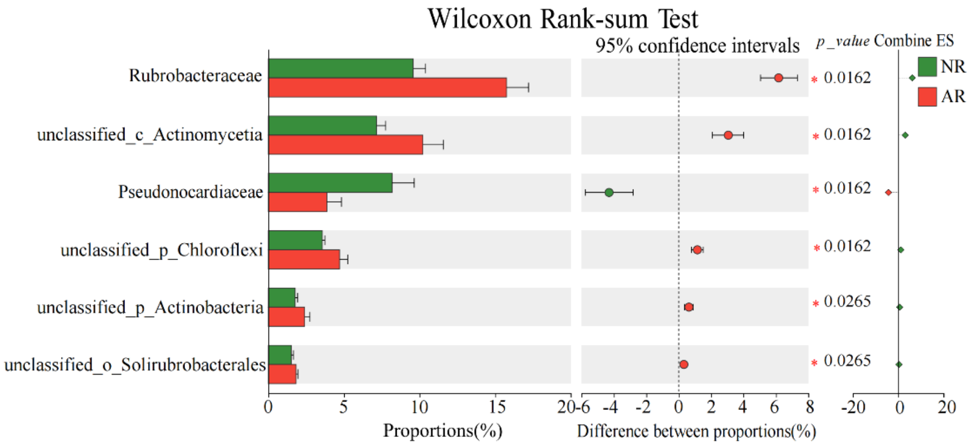

Figure S3. Microbial families with significant differences under different restoration strategies. NR, natural recovery; AR, artificial restoration. * indicates significant differences at $p < 0.05$.

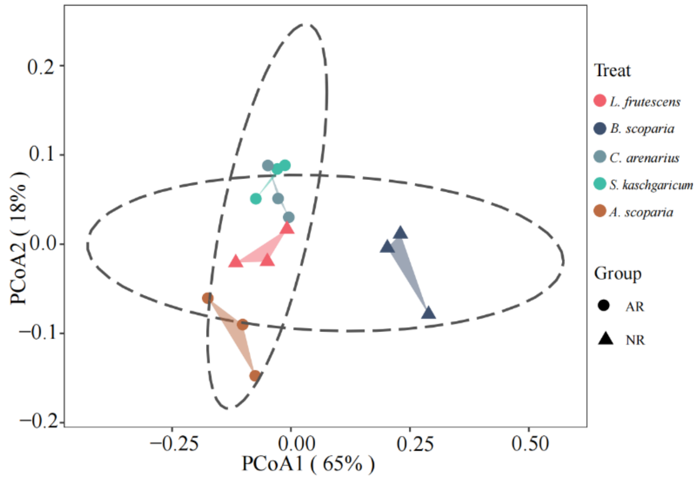

Figure S4. Classification level of microbial family PCoA. NR, natural recovery; AR, artificial restoration. *A. Scoparia*, *Artemisia scoparia*; *S. Kaschgaricum*, *Seriphidium kaschgaricum*; *C. Arenarius*, *Ceratocarpus arenariu*; *B. Scoparia*, *Bassia scoparia*; *L. Frutescens*, *Leucophylum frutescens*.

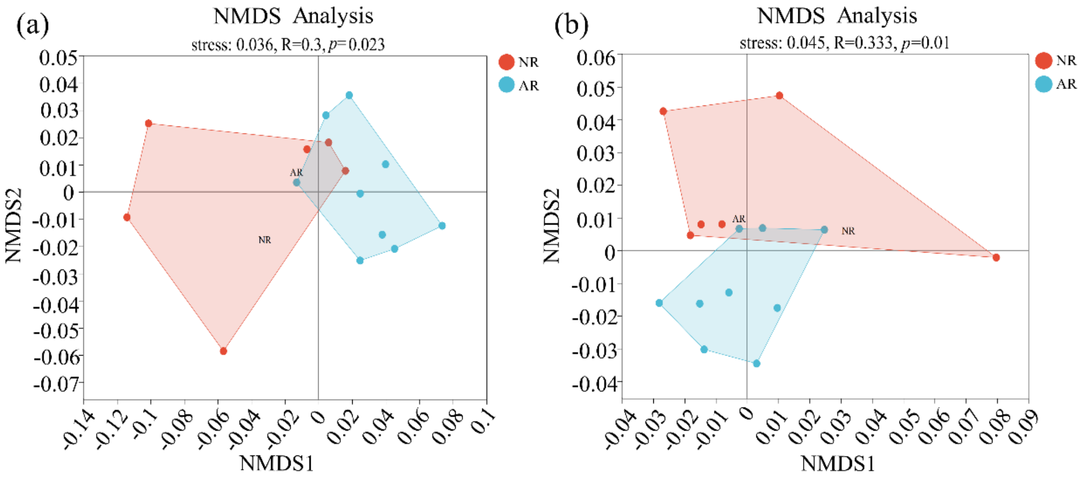

Figure S5. NMDS analysis on the carbon decomposition genes (**a**) and carbon fixation genes (**b**) difference under different restoration strategies. NR, natural recovery; AR, artificial restoration.

Table S1 Soil Variables and Detection Methods

Soil variables	Detect method
pH (potential of hydrogen)	pH meter method
EC (electrical conductivity)	electric conductivity method
SC (salt content)	gravimetric method
SOC (soil organic carbon)	Potassium dichromate dilution heating method
NO_3^--N (nitrate nitrogen)	ultraviolet spec- trophotometry
NH_4^+-N (ammonium nitrogen)	colorimetric blue indoxyl method
TP (total phosphorus)	molybdenum antimony colorimetric method
AP (available phosphorus)	molybdenum antimony sodium bicarbonate anti colorimetric method
Urease activity	sodium hypochlorite phenol colorimetric method
Phosphatase activity	disodium phenyl phosphate colorimetry

Table S2 Plant-tissue Variables and Detection Methods

Plant-tissue variables	Detect method
PTN (plant tissue total nitrogen)	sulfuric acid-hydrogen peroxide digestion method
PTP (plant tissue total phosphorus)	sulfuric acid-hydrogen peroxide digestion method
POC (plant tissue organic carbon)	potassium dichromate-sulfuric acid oxidation colorimetric method
Cellulase activity	3,5-dinitrosalicylic acid colorimetric method
Catalase (CAT) activity	Ultraviolet spectrophotometry
Soluble sugar	Anthrone-sulfuric acid spectrophotometr
Starch	3,5-dinitrosalicylic acid colorimetric method

Table S3 The function, gene and KO of carbon decomposition

Function	gene	KO
Starch	*alpha-amylase*	K01176
	alpha-amylase	K07405
	alpha-amylase	K05343
	pullulanase	K01200

	neopullulanase	K01208
	neopullulanase	K21575
	glucoamylase	K01178
	1,4-alpha-glucosidase	K21574
	alpha-glucosidase	K01187
	isoamylase	K01214
	1,4-alpha-glucan branching enzyme	K00700
	glycogen debranching enzyme	K02438
	glycogen phosphorylase	K00688
	glycogen phosphorylase/synthase	K16153
	4-alpha-glucanotransferase	K00705
	4-alpha-glucanotransferase	K22451
	oligo-1,6-glucosidase	K01182
Cellulose	*beta-glucosidase*	K05349
	beta-glucosidase	K05350
	endoglucanase	K01179
	endoglucanase	K20542
	cellobiose phosphorylase	K00702
	6-phospho-beta-glucosidase	K01222
	6-phospho-beta-glucosidase	K01223
	1,3-beta-glucosidase	K01210
	1,4-beta-cellobiosidase	K19668
	lytic cellulose monooxygenase	K22033
Lignin	*galactose oxidase*	K04618
	laccase	K00421
	multicopper oxidase	K22552
	polyphenol oxidase	K00422
	tyrosinase	K00505
	catalase	K03781
	catalase-peroxidase	K03782
	non-heme chloroperoxidase	K00433
	cytochrome c peroxidase	K00428
	glutathione peroxidase	K00432
	NADPH:quinone reductase	K00344
	NADPH:quinone reductase	K00359
	glutathione reductase (NADPH)	K00383
Pectin	*pectate lyase*	K01728
	pectate lyase	K22539
	pectinesterase	K01051
	polygalacturonase	K01184
	galacturan 1,4-alpha-galacturonidase	K22933
	alpha-L-rhamnosidase	K05989
	rhamnogalacturonan acetylesterase	K15530
	rhamnogalacturonan endolyase	K18195

	rhamnogalacturonan endolyase	K18197
	rhamnogalacturonan exolyase	K18198
	unsaturated rhamnogalacturonyl hydrolase	K15532
Hemicellulose	*non-reducing end alpha-L-arabinofuranosidase*	K20844
	alpha-L-arabinofuranosidase	K01209
	xylose isomerase	K01805
	endo-1,4-beta-xylanase	K01181
	arabinoxylan arabinofuranohydrolase	K15921
	arabinan endo-1,5-alpha-L-arabinosidase	K06113
	beta-mannosidase	K01192
	mannosyl-oligosaccharide alpha-1,2-mannosidase	K01230
	alpha-mannosidase	K01191
	beta-1,2-mannosidase	K21065
	mannan endo-1,4-beta-mannosidase	K01218
	mannan endo-1,4-beta-mannosidase	K19355
	xylan 1,4-beta-xylosidase	K01198
	xylan 1,4-beta-xylosidase	K22268
	beta-glucuronidase	K01195
	acetylxylan esterase	K05972
	alpha-galactosidase	K07406
	alpha-galactosidase	K07407
	beta-galactosidase	K01190
	beta-galactosidase	K12308
	feruloyl esterase	K09252
	alpha-glucuronidase	K01235
	alpha-L-fucosidase	K01206
	alpha-L-fucosidase 2	K15923
	alpha-D-xyloside xylohydrolase	K01811
	arabinogalactan endo-1,4-beta-galactosidase	K01224
	carboxylesterase	K03928
	levanase	K01212
	endo-1,3(4)-beta-glucanase	K01180
Chitin	*alpha-N-acetylglucosaminidase*	K01205
	chitinase	K01183
	beta-N-acetylhexosaminidase	K01207
	hexosaminidase	K12373
	N,N'-diacetylchitobiose phosphorylase	K18675
	alpha-N-acetylglucosaminidase	K01205
	chitinase	K01183
	beta-N-acetylhexosaminidase	K01207
	hexosaminidase	K12373
	N,N'-diacetylchitobiose phosphorylase	K18675
Aromatic	*methylisocitrate lyase*	K03417
	isocitrate lyase	K01637

malate synthase	K01638
2-isopropylmalate synthase	K01649
limonene-1,2-epoxide hydrolase	K10533
vanillate monooxygenase	K03862
vanillin dehydrogenase	K21802

Table S4 The function, gene and KO of carbon fixation

Function	gene	KO
Calvin cycle	*rbcL*	K01601
	rbcS	K01602
	gap2	K00150
	GAPDH	K00134
	ALDO	K01623
	FBA	K01624
	FBP	K03841
	glpX	K02446
	glpX-SEBP	K11532
	fbp-SEBP	K01086
	EC 2.2.1.1	K00615
	rpiA	K01807
	rpiB	K01808
Arnon–Buchanan cycle	*porA*	K00169
	porB	K00170
	porD	K00171
	porC	K00172
	por	K03737
	pps	K01007
	ppdK	K01006
	ppc	K01595
	pycA	K01959
	pycB	K01960
	PC	K01958
	mdh	K00024
	E4.2.1.2A	K01676
	E4.2.1.2B	K01679
	E4.2.1.2AA	K01677
	E4.2.1.2AB	K01678
	sdhA	K00239
	sdhB	K00240
	sdhC	K00241
	sdhD	K00242
	frdA	K00244
	frdB	K00245
	frdC	K00246

	frdD	K00247
	frdA	K18556
	sucD	K01902
	sucC	K01903
	korA	K00174
	korB	K00175
	korC	K00177
	korD	K00176
	IDH1	K00031
	ACO	K01681
	acnB	K01682
	aclA	K15230
	aclB	K15231
	ccsA	K15232
	ccl	K15234
Incomplete reductive citrate cycle	*porA*	K00169
	porB	K00170
	porD	K00171
	porC	K00172
	pycA	K01959
	pycB	K01960
	mdh	K00024
	E4.2.1.2AA	K01677
	E4.2.1.2AB	K01678
	sucD	K01902
	sucC	K01903
	korA	K00174
	korB	K00175
	korC	K00177
	korD	K00176
	tfrA	K18209
3-hydroxypropionate bicycle	*E4.2.1.2B*	K01679
	sdhA	K00239
	sdhB	K00240
	sdhC	K00241
	accB	K02160
	accC	K01961
	accA	K01962
	accD	K01963
	K14469	K14469
	K15052	K15052
	MCEE	K05606
	MUT	K01847
	E5.4.99.2A	K01848

	E5.4.99.2B	K01849
	mcl	K08691
	mch	K14449
	mct	K14470
	meh	K09709
Dicarboxylate–4-hydroxybutyrate cycle	*porA*	K00169
	porB	K00170
	porD	K00171
	porC	K00172
	pps	K01007
	ppc	K01595
	mdh	K00024
	E4.2.1.2AA	K01677
	E4.2.1.2AB	K01678
	sdhA	K00239
	sdhB	K00240
	sdhC	K00241
	sucD	K01902
	sucC	K01903
	K14465	K14465
	abfD	K14534
	K15016	K15016
	ACAT	K00626
4-hydroxybutyrate cycle	*MCEE*	K05606
	E5.4.99.2A	K01848
	E5.4.99.2B	K01849
	K15019	K15019
	K15020	K15020
	K14465	K14465
	abfD	K14534
	K15016	K15016
	ACAT	K00626
Wood–Ljungdahl pathway	*fdhA*	K05299
	fdhB	K15022
	fdhF	K22015
	fhs	K01938
	folD	K01491
	fchA	K01500
	metF	K00297
	acsE	K15023

Table S5 Differences in soil physical and chemical properties under different restoration strategies

Soil variables	NR	AR
pH	8.05±0.01^{a}	8.06±0.03^{a}
EC (μS·cm^{-1})	1211.5±76.15^{a}	1045.89±30.92^{b}
SC (mg·L^{-1})	60.7±3.42^{a}	52.1±1.64^{b}
SOC (g·kg^{-1})	16.08±0.19^{a}	16.52±1.28^{a}
TP (g·kg^{-1})	0.30±0.08^{a}	0.35±0.04^{a}
AP (mg·kg^{-1})	30.57±6.29^{a}	36.59±0.99^{a}
NO_3^--N (mg·kg^{-1})	1.35±0.14^{b}	7.10±3.53^{a}
NH_4^+-N (mg·kg^{-1})	1.85±0.14^{a}	1.03±0.17^{b}
Phosphatase activity (μmol g^{-1} h^{-1})	56.24±6.06^{a}	73.13±23.56^{a}
Urease activity (μg N g^{-1} h^{-1})	1.08±0.18^{a}	1.02±0.12^{a}

Soil variables: pH, soil pH; EC, electrical conductivity; SC, salt content; SOC, soil organic carbon; TP, total phosphorus; AP, available phosphorus; NO_3^--N, nitrate nitrogen; NH_4^+-N, ammonium nitrogen; Phosphatase, phosphatase activity; Urease, urease activity. Peer lowercase letters indicated significant difference ($p < 0.05$). NR: natural recovery; AR: artificial restoration.

Table S6 Differences in plant-tissue physical and chemical properties under different restoration strategies

Plant-tissue variables	NR	AR
PTN (mg·kg^{-1})	0.95±0.29^{b}	1.73±0.73^{a}
PTP (mg·kg^{-1})	0.04±0.02^{a}	0.05±0.01^{a}
POC (g·kg^{-1})	482.89±98.99^{a}	429.29±39.23^{a}
Cellulase activity (μmol glucose g^{-1}·h^{-1})	23.08±7.78^{a}	16.77±4.32^{a}
CAT (μmol H_2O_2 g^{-1}·min^{-1})	5.33±0.65^{a}	4.76±2.32^{a}
Soluble sugar (%)	4.95±0.15^{a}	5.34±0.57^{a}
Starch (%)	17.83±0.54^{b}	19.25±2.09^{a}

Plant-tissue variables: CAT, catalase activity; Cellulase, cellulase activity; POC, plant tissue organic carbon; PTP, plant tissue total phosphorus; PTN, plant tissue total nitrogen; Soluble sugar, plant soluble sugar; Starch, plant starch. Peer lowercase letters indicated significant difference ($p < 0.05$). NR: natural recovery; AR: artificial restoration.

Table S7 *P*-values for differences in relative abundances of microbial domains between the two restoration strategies.

Name	Bacteria	Archaea	Eukaryota	Viruses	Unclassified
p	0.953	0.1407	0.1407	0.1407	0.1407

Table S8 PERMANOVA results (permutational multivariate analysis of variance) comparing

microbial community structure at the family level between the two restoration strategies.

Name	R^2	Pr(>F)
Restoration	0.23664302294	0.028

Table S9 The KO, gene abbreviation and full name of carbon fixation.

KO	Gene abbreviation	Full name
K01681	*ACO*	*aconitate hydratase*
K00626	*ACAT*	*acetyl-CoA acetyltransferase*
K01006	*ppdK*	*pyruvate orthophosphate dikinase*
K01595	*ppc*	*phosphoenolpyruvate carboxylase*
K01903	*sucC*	*succinyl-CoA synthetase beta subunit*
K01902	*sucD*	*succinyl-CoA synthetase alpha subunit*
K00239	*sdhA*	*succinate dehydrogenase flavoprotein subunit*
K00240	*sdhB*	*succinate dehydrogenase iron–sulfur subunit*
K00174	*korA*	*2-oxoglutarate: ferredoxin oxidoreductase alpha subunit*
K01848	*E5.4.99.2A*	*methylmalonyl-CoA mutase subunit*
K05606	*MCEE*	*methylmalonyl-CoA epimerase*
K01961	*accC*	*acetyl-CoA carboxylase biotin carboxylase subunit*
K00297	*metF*	*5,10-methylenetetrahydrofolate reductase*
K01491	*folD*	*methylenetetrahydrofolate dehydrogenase / methenyltetrahydrofolate cyclohydrolase*
K02446	*glpX*	*fructose-1,6-bisphosphatase IIsedoheptulose-1,7-bisphosphatase*
K00615	*EC 2.2.1.1*	*transketolase*
K01601	*rbcL*	*ribulose-1,5-bisphosphate carboxylase/oxygenase large subunit*
K01602	*rbcS*	*ribulose-1,5-bisphosphate carboxylase/oxygenase small subunit*
K15230	*aclA*	*ATP-citrate lyase alpha subunit*

K15231	*aclB*	*ATP-citrate lyase beta subunit*
